# “Your Thoughts are (were) Free!“: Brain-Computer-Interfaces, Neurofeedback, Detection of Deception, and the Future of Mind-Reading

**DOI:** 10.1007/s10484-024-09648-z

**Published:** 2024-06-14

**Authors:** Niels Birbaumer

**Affiliations:** Department of Medical Psychology and Behavioral Neurobiology, University of Tübingen, Silcherstrasse 5, 74072 Tübingen, Germany

**Keywords:** Brain-computer-interfaces, Mind Reading, Neurofeedback, Amyotrophic Lateral Sclerosis, Paralyis, Ethical Aspects

## Abstract

This review describes the historical developement and rationale of clinically relevant research on neurophysiological „mind reading“ paradims: Brain- Computer-Interfaces, detection of deception, brain stimulation and neurofeedback and the clinical applications in drug resistant epilepsy, chronic stroke, and communication with paralyzed locked-in persons. The emphasis lies on completely locked-in patients with amyotrophic lateral sclerosis using non-invasive and invasive brain computer interfaces and neurofeedback to restore verbal communication with the social environment. In the second part of the article we argue that success and failure of neurophysiological „mind reading“ paradigms may be explained with a motor theory of thinking and emotion in combination with learning theory. The ethical implications of brain computer interface and neurofeedback approaches, particularly for severe chronic paralysis and loss of communication diseases and decisions on hastened death and euthanasia are discussed.

*"Thank God there are still doubters and those who have the urge to procrastinate. As if everyone who is willing to take the plunge and make demands would be a role model for us and a good fellow citizen for the country to which he belongs. Exactly not! And the unfinished are more finished than the finished, and the unusable are often much more useful than the useful, and besides, not everything needs to be available for use immediately or in the shortest possible time. A certain human luxury lives on happily even in our times, and a society falls into the hands of the devil, which wants to eradicate every comfort and ease."*

Robert Walser (1926)

Robbers (Räuber) Novel ca 1925/2022

“*Whatever interferes with tentative movements will inhibit all thinking*”.

Margaret F. Washburn (1928)

## Introduction

The Brain-Computer-Interface (BCI) literature is dominated by technical and methodological descriptions, the progress in engineering and artificial intelligence (AI) algorithms for non-invasive BCIs as well as electrode-construction and testing for the invasive BCI. Very few contributions report on reproducable and clinically relevant applications. For non-invasive electroecephalography (EEG) and near infrared spectroscopy (NIRS) applications, healthy subjects are mainly asked to move wheel-chairs or other assistive technology, using a BCI. Still high error rates of noninvasive approaches in natural environments (e.g. Ramos-Murguialday et al., 2019) complicate clinical applications in neuroprosthetic rehabilitation. Invasive approaches provide more accuracy but are often difficult to implement in the patients’s natural environment and have so far rarely been used in completely locked in states.

Here we provide a narrative review with a focus on clinical problems where no other alternative other than BCI technology is available for the particular patient population or, as in chronic stroke, a clear superiority of the BCI approach over traditional treatment was observed. Because lie detection with brain signals and non-invasive brain stimulation are based on the same assumptions as BCI applications and often preceeded BCI research or followed BCI-applications, we include these brain technologies in our review and theoretical framework.

Instead of emphasising the technical engineering and algorithm-dominated approaches, we try to embed BCI-applications in a unifying neuroscientific and neurobehavioral theoretical framework and discuss some specific ethical standards in order to guide future development into clinically and ethically acceptable directions. We focus mainly on the work of our group but include important other approaches, where apprropriate.

## State of Research

### Brain-Computer-Interfaces

Brain-Computer-Interfaces (BCI), also called Brain-Machine-Interfaces (BMI), are technical sytems, which transfer the brain activity of humans or animals *directly* to computers or machines. „Directly“ means that the motor system and the peripheral nervous system are bypassed and the brain activity (mostly electrical, magnetic or metabolic) can activate or de-activate, i.e. switch on or off computers or machines via a corresponding interface. In the standard case, which was the only one possible until the development of BCIs in the 20th century, the human or animal activated or blocked (inhibited) its motor system (behavior) with brain signals and thus controlled its environment indirectly from the brain via the *mediation* of the motor system with an actual motor response. The perception of the feedback of these actions, which also include speech and communication, determines whether we maintain or abandon these actions (behaviors) in the future.

In this respect, the basic idea of the BCI cannot be distinguished from the usual evolutionary pathway from the brain command to the behavior; it differs only such that behavior (movement, language), i.e. the motor mediation, is no longer necessary to manipulate the environment (feedback), but the corresponding brain commands (mostly electromagnetic or neurometabolic changes from brain cells and cellular processes of the neurons and axons) can directly control the environment (machines and computers) and thus also the *feedback* from the environment via the technical interfaces. In both cases, the use of BCI-controlled behavior and the usual case of motivated behavior - which has been common throughout evolution - we are not aware of the brain processes that take place and how they are transmitted to muscles or machines. The organisms learn to change and control their brain processes indirectly via the achieved feedback: since the brain cells have *no receptors for their own electromagnetic activity*, we and animals can neither consciously nor unconsciously perceive the activity changes of the neurons. The same is true for neurofeedback which preceded BCI-research historically. However, the lack of access to consciousness does not mean that we are unable to influence and consciously control the neural processes underlying our behavior (and thinking) via the perception of the changes achieved in the environment. The results of neurofeeback research over the last 60 years show that we (by “we” we always mean higher animal species) can develop a conscious feeling in the form of perceptual changes for some brain processes (e.g. brain waves as recorded with the EEG or blood flow changes recorded with magnetic resonance imaging (MRI) or NIRS after prolonged training with positive feedback of our own electrical or metabolic brain activity. EEG, MRI and NIRS changes are always the result of the acivity of millions of neurons. Learning to perceive activity from one or a few neurons is probably not possible, although the last word on this has not yet been said experimentally (see below the invasive BCI realised in patient F., Chaudhary et al., 2022). Without falling into questionable evolutionary explanations of purpose (“perception of bodily processes of the internal organs and the brain is evolutionarily directed against survival”), it would probably make little sense and would completely block our behavior and selective attention if we could consciously perceive our electromagnetic changes in the brain that produce thoughts and behaviors in daily life!

### Lie Detection

Methods for “detection of deception”, as it is somewhat euphemistically called in the professional literature and by criminologists, have emerged from psychophysiological research earlier than BCI technology. But both are based on the same psychophysiological processes and the same scientific and logical principles: both strive to make the normally hidden (“covert”) thoughts and their physiological correlates perceptible, to “read” thoughts. In those lie detectors that use brain processes as a classification measure, the motor (behavior, speech) and peripheral physiological changes (e.g., blushing or sweating) are disregarded, just as in BCI, and the thoughts (electrical brain processes) are made directly accessible with a polygraph or other recording instruments (Rosenfeld, 2018). In contrast to BCI, however, the polygraph assumes that the person concerned “wants” to make only certain, specific thoughts perceptible, but does not intend to disclose others. But it is exactly these thoughts that the questioner(s), the experimenter(s) would like to “read”. Since all thoughts are generally based on the same neuroelectrical processes (neuro-electrical spikes and graded synaptic potentials of neurons), it is only a matter of distinguishing a certain type, a specific category of thoughts - and thus neural processes - from another type, i.e. “true” from “false” (concealed) thoughts. Thus, a problem of specific differentiation of a physiological measure (correct-false) is added to the BCI thought-reading principle. Otherwise, however, it is the same concern as with BCI neuroprostheses, e.g., to transfer a movement impulse and movement plan (“true”), i.e. in a paralyzed human or paralyzed animal directly from the brain to a prosthesis attached to the hand and to suppress a competing thought or intention(“false”). This task is possible to attain and is realized under certain circumstances (see below).

Nowadays *artificial Intelligence (AI)* algorithms are increasingly used for “criminological” truth detection. Most of them try to distinguish the “truth” from the “lie” based on facial expression, as well as the linguistic answers and the physiological reactions of the “delinquents” and suspects. However, since AI is always based on the input of large amounts of stored data from many consciously or unconsciously biased persons (or the organizations on whose data the algorithms depend), one must be very critical of these classification attempts; they cannot use more than the classified input, if the stored input is biased, the end result is also biased.

The recent political, economic and scientific enthusiasm for AI may end in a humanitarian, ecological and political disaster. It is not AI that may cause this disaster and is already doing so, but ignorance and inertia of ingrained and “cherished“(overlearned) habits and behaviors. Just as we cannot give up the car today because urban architecture is based on roads and we can only reach vital destinations by car, we may not be able to find our way without the AI-controlled orientation systems and our supply with the necessary money from banks may no longer be possible without AI algorithms, and so on. Politicians are energetically pursuing the supposedly important and useful „digitalization of the citizens“ without having any idea of the dependencies they are driving themselves and the citizens into.

Historically, the recognition of false statements and thoughts (“lies”) has been the subject of intense “experiments” since the ancient Egyptians. The heart was removed from the dead pharaohs as the presumed seat of the soul and placed on a kind of scale. The dead person was asked about their good or bad deeds: if the heart became heavier after a question, this indicated a bad deed during their lifetime and prevented the favor of positively tuned deities.

The first physiologically based lie detectors were used in China around 1000 BC: the accused had to fill their mouth with rice and spit it out; if it was dry, they had lied and were guilty, as fear makes the mouth dry by inhibiting saliva production. This corresponds to the logic of modern lie detectors.

In the Christian Occident up to the seventeenth century, “God’s Judgements” were highly popular: the interrogated and usually accused suspects, often female and prejudged as a witch or heretic, received, for example, a red-hot iron rod in the hand, if the burning failed, they had told the truth and were pardoned, but this occurred only with saints. The variations of these judgments of God all ended with the death by annihilation of the accused.

The Chinese method was taken up in the nineteenth century by Cesare Lombroso („Genio e follia“,“Genius and Madness”, 1864; L’Uomo delinquente, 1876). Lombroso measured blood pressure changes and respiration instead of a dry mouth, in 1920 skin resistance (measurement of sweat gland activity, not sweat! ) was added, and Lombroso assumed that an increase of arousal in an interview situation would indicate that the person lied. However, it was soon recognized that sweat gland activity correlated only nonspecifically with stress, and was not specific for lying. Only the introduction of questioning and experimental testing with the *Guilty Knowledge Test (GKT)* by the psychophysiologist David Lykken (Lykken, 1998) improved the poor validity of the lie detector: Only if the questioner (interrogator) knows the course of events very precisely and asks many control questions in multiple choice question mode does the lie detector work - sometimes even up to 100% correct. For example, some questions are: “The crime was committed with an a.) yellow, b.) gray, c.) black, d.) silver revolver”. And/or " The room of the crime was painted a.) green, b.) yellow, c.) red d.) white,” and so on. Then, around 1990, instead of skin resistance (activity of sweat glands), brain-evoked EEG potentials were introduced by Peter Rosenfeld and Emanuel Donchin (Rosenfeld, 2018): for the relevant words an increase in brain potential due to the attentional focus already between 200 and 300 milliseconds was measured. Since it is assumed that it is impossible to activate lie control mechanisms such as distraction or voluntary excitement or thought control of the relevant physiological measures in such a short time, the amplitude of the measures (evoked brain potentials recorded with EEG) increased the reliability significantly in many such questions of the GKT. As with brain evoked potentials the exact knowledge of the event is a prerequisite for the construction of the correct multiple choice questions. BCIs in combination with AI algorithms will allow a clear separation of lies and truth from corresponding changes of brain activity, the lie detector can advance to a “thought detector” (see below).

### History of BCI and Brain Stimulation

#### BCI

The term BCI as we use it today was first proposed by Jaques Vidal in 1973 and Vidal was also one of the first to use EEG oscillations, especially the alpha rhythm of 8–13 Hz to control displays (computer cursors) and light signals on a screen. However, the initial research for non-invasive BCI had been realized much earlier in 1960 by Joe Kamiya under the term biofeedback (later neurofeedback), who trained his subjects at the University of California, San Francisco to control their own alpha rhythm by playing them a light or sound signal whenever alpha waves were measured in the EEG (Kamiya, 2011). Most subjects indicated that they used different imaginations to manipulate the feedback signals. This laid the technical foundation for BCI and neuroprostheses, because instead of signals on a screen, one can control any external device connected to a technical interface that converts brain signals into device signals.

The development of such *non-invasive* BCIs and neurofeedback controlled by brain waves derived from electrodes on the human scalp, had first been described by Hans Berger, the Jena-based discoverer of human EEG in 1929 (Berger, 1929) and shortly thereafter by his “competitor”, the Tyrolean psychologist Hubert Rohracher, but this was not called BCI but viewed as a possiblity to use EEG for practical purposes (Rohracher, 1937).

**Fig. 1:**
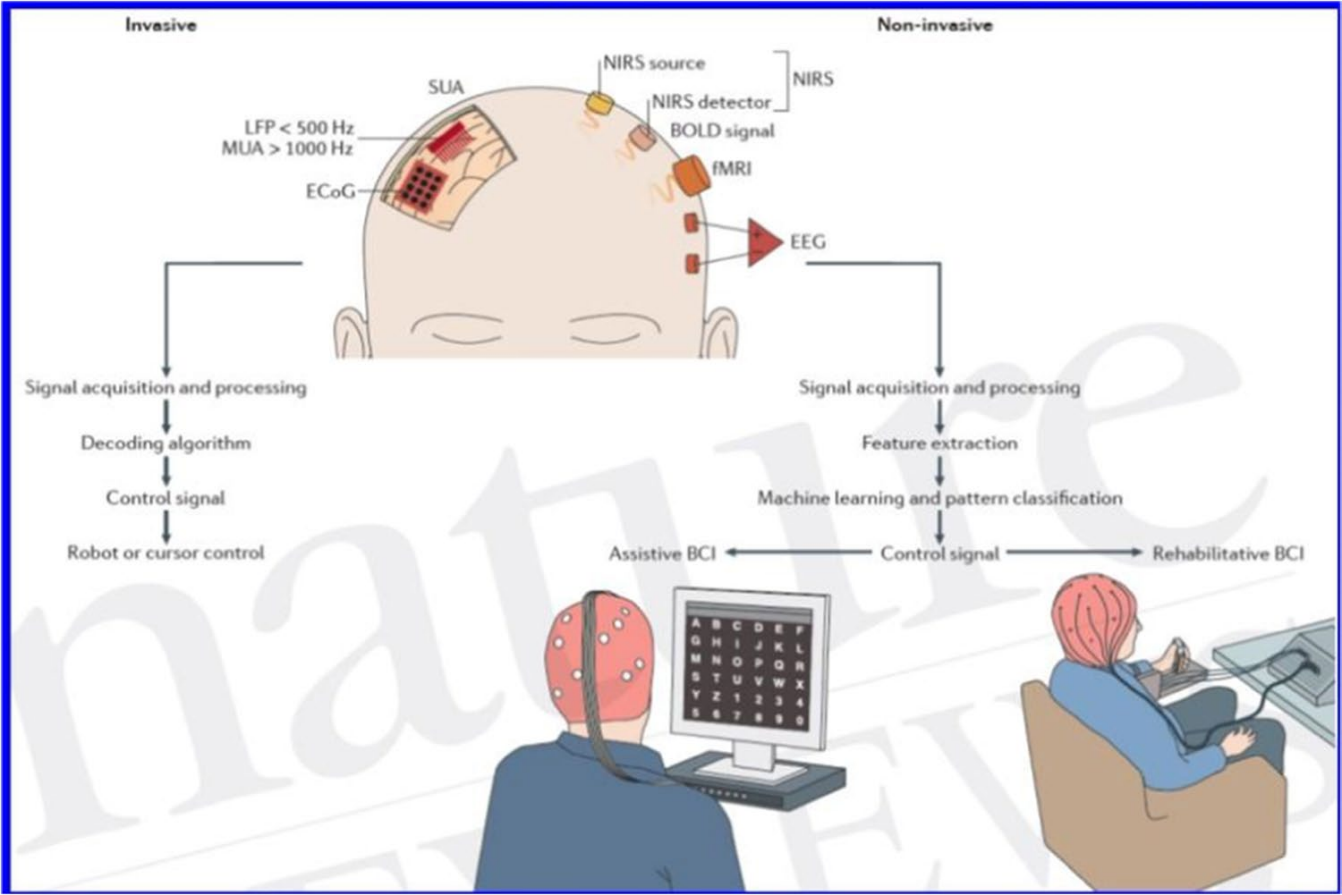
Brain-Computer-Interfaces (BCI).  Top: Invasive (left) and non-invasive (right) BCIs. Below: Letter selection from a computer menu with brain potentials left and prosthesis control on the right. Abbreviations: ECoG, electrocorticogram. MUA, multiple unit acivity. LFP, local field potential outside of cells. SUA: single unit activity. NIRS, near infrared spectroscopy. BOLD, blood oxygen level dependent: effect to measure neural activity indirectly in fMRI. fMRI, functional magnetic resonance imaging. EEG, electroencephalography. (From Chaudhary et al., Nature Reviews Neurology, 2017; with kind permission)

Research on *invasive BCIs*, in which feedback signals or switching signals for the computer originate from electrodes implanted in the brain in close proximity to neurons or directly into neurons with microelectrodes, has been somewhat delayed (see Fig. 1 - a depiction of possible BCIs).

Although he did not yet call it BCI, the first experiment on it was by the German-American physiologist Eberhard Fetz (1969) on a monkey that learned to manipulate the firing of the cells in the cerebral cortex: the monkey was motivated for phases of rise and fall of cellular responses with immediate reward (fruit juice or food). Fetz emphasized that the decisive factor for learning brain control is *operant conditioning*, experimentally described in the psychology of learning, especially by B. F. Skinner. The reward can also be replaced by a feedback signal or a prosthetic or robotic movement, as long as this has a rewarding character, as is usually the case with neurofeedback and BCI (if one can suddenly move a paralyzed hand again by one’s own will (in imagination or in reality), this usually means an enormous psychological reward and satisfaction for the patient! ). Invasive BCI research in the first two decades of ist existence was realized completely separately from the non-invasive BCI and neurofeedback research.

It stimulated the development of microelectrode arrays, in which hundreds of microelectrodes are combined into a bundle and can be surgically implanted into the human cortex with relative safety. These bundles (arrays) of hundreds of tiny metal electrodes are smaller than a one-cent piece and record the electrical discharges (potentials) of several hundred neurons.

Today, such arrays are commercially available, the best studied and FDA- and EU approved array “Neuroport”, tested on many patients especially by John Donoghue and his group at Brown University, is used today in neuroprosthetics as well as for BCI communication of totally paralyzed patients (Birbaumer and Hochberg, 2018; Chaudhary et al., 2022).

Only in recent years did collaboration between researchers in the non-invasive and invasive BCI traditions occur after it was realized that, above all, knowledge and use of principles of learning psychology and neurofeedback are necessary for the application of invasive BCIs as well and, conversely, that in some applications non-invasive BCIs are less expensive and more useful for patients (e, g., stroke). In other applications, such as verbal communication with the completely paralyzed, invasive microelectrode systems achieve even better results (see below).

The multi-electrode BCI developed by the company Neuralink (Musk & Neuralink, 2023) uses thousands of microelectrodes. Musk is primarily interested in commercial use with healthy people, but so far the Neuralink electrodes have only been used to realize neuroprosthetics experiments in animals and one paraplegic person. Nevertheless, these electrodes are much smaller than all other existing electrodes at least for the cortex. They are not suitable for deeper brain regions due to their size and material composition. BCI applications that aim to visualize and modify emotional-motivational processes from deep brain regions are thus ruled out with presently available electrode arrays except for those used for deep brain stimulation and recordings in patients with Parkinson’s disease.

### Brain Stimulation

The development and future of BCI is closely tied to the history of brain stimulation in humans. Brain stimulation preceded the development of BCI by decades. That specific psychological processes can be inferred from electrical and magnetic brain activity or metabolic parameters (e.g., blood flow, cortisol changes) was known long before the existence of BCIs through the effects of electrical stimulation of the brain in animals. The largely disastrous consequences of psychosurgery in the mid-20th century, especially leukotomy (lobotomy) also accelerated the development of brain stimulation when the permanent negative consequences of the destruction of brain matter without sufficient knowledge of its functions became clear. Non-invasive brain stimulation outside the skull received its impetus as a result. Psychosurgery was further discredited by fanatic brain surgeons and psychiatrists such as the Texan Robert Heath, who tried to „eliminate“ homosexuality with chronic brain stimulation of, among other things, the reward system during (forced) heterosexual sexual acts, thus triggering the later suicide of the” patient”.

Since electrical brain stimulation causes only small and very localized damage to brain matter and one can remove and turn off the stimulation electrodes, it later became an alternative to lesioning neurosurgical treatment for brain dysfunction (especially motor neurological diseases such as Parkinson’s disease) and its success has taught us where in the brain regions relevant for certain behaviors are located and which are critical for “reading out” cognitive, emotional, motor and sensory performance in BCI.

This principle was clear already in the antiquity, when the Roman “researcher” Scribonius Largus recommended 47 after Christ touching electric fish as a method of therapy for a number of diseases (Sribonius 1529/2011). However, due to the view, triggered by the Egyptians and adopted by Aristotle, that the heart was a carrier of psychological (soul) functions, research and knowledge of brain functions was blocked until the Renaissance, although Arabic-Islamic-Jewish science in Europe from the 9th century until the proto-Renaissance emperor Frederick II in southern Italy around 1250, returned to the brain as a “soul carrier”. But, it could not prevail through the papal ideology committed to Aristotle. Only the experiments of Luigi Galvani and his nephew Giovanni Aldini in the middle of the 18th century brought the experimentally demonstrated breakthrough for electrical brain stimulation, initially on animals.

The dramatic success of deep brain stimulation in Parkinson’s disease, which was developed by Alim Benabid in Grenoble in the 1960s and 1970s (Benabid, 2003), paved the way for the widespread use of invasive and noninvasive brain stimulation in other neurological and mental disorders.

Finally, remembering the many anecdotal experiments since the use of the electric fish in ancient times, there was a still ongoing revival of non-invasive brain stimulation as well, including the development of direct current (d.c.) stimulation of the human brain with electrodes attached to the skull and scalp, now called transcranial direct current stimulation (tDCS; Elbert et al., 1981). By applying a direct current of a few milli-amperes to certain parts of the head penetrating skin and bone, even strong willful impulses for certain hand movements could be manipulated without conscious intention (and presumably also *against* existing intentions), without the test person noticing anything. The current direction of the imperceptible stimulation current determined the action, not the intention, the will of the subject.

Transcranial magnetic stimulation (TMS), in which an eight-shaped coil is used to induce fast (10–50 Hz) magnetic stimulating or slow (below 5 Hz) inhibitory effects in the underlying brain tissue, has become an indispensable standard procedure in clinical neurology and psychiatry. For example, significant effects have been reported for major depression (Brini et al., 2023).

### Clinical Applications of Neurofeedback and BCI

#### Drug-restistant Epilepsies

Epilepsies have been a “magic” disease for thousands of years with many ingenious affected representatives (Dostojevsky, Van Gogh, allegedly Caesar, Charles III “The Fat One”, Pascal, Beethoven and others) and an excellent neurophysiological model for the study of excitation regulation in the brain and thus also for cognitive processing. Before the actual seizure, no matter if it is only a short absence or a motor seizure with fall, convulsions and unconsciousness, there is already an increase of excitation in the affected brain tissue in one local circumscribed part of the brain (e.g. psychomotor attacks, motor stereotypies) or in the whole cortex (generalized seizure); a state often called „aura“ (from the Greek, meaning breath). Since the introduction of antiepileptic drugs in the last century, however, auras have become very rare, since the drugs already prevent the increase in brain-excitation and thus also eliminate the perception of an aura. Many epileptic patients could either control and prevent the seizures after the first signs with psychological strategies (relaxation, odor stimuli, avoidance of flickering light and of certain food and stress). 30% of all patients do not respond to drugs. These are mainly persons with secondary generalized epilepsies, which spread from one brain region to all cortex regions sometimes over longer periods of time and can lead to the death of neurons and, in extreme cases, entire brain regions with severe cognitive deficits if seizures occur frequently, and were often heralded by auras. If the seizure with hyperexcitation originates from one area of the brain (like the hippocampus of one or both temporal cerebral hemispheres), seizures can often be terminated with neurosurgical removal of this brain region. However, many patients are not operable because vital and cognitively important brain regions are affected (e.g. language in left hemisphere). We (Rockstroh et al., 1989; Kotchoubey et al., 2001) have therefore imitated the mode of action of drugs in a psychological procedure and developed a neurofeedback method for learning seizure control and tested it on many untreatable, drug-resistant epileptic patients.

**Fig. 2:**
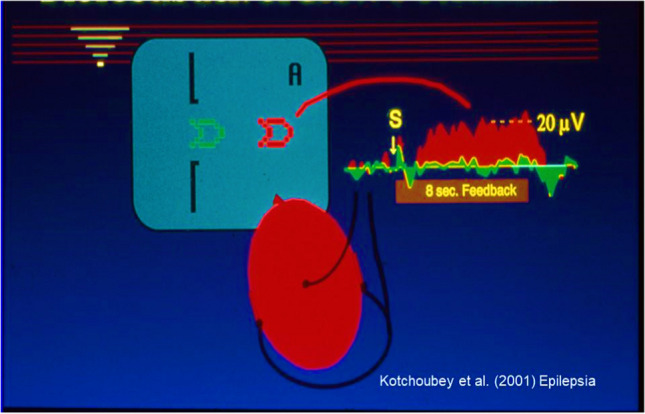
Neurofeedback of slow cortical potentials (SCP) in drug-resistant epilepsy. The left upper side of the figure shows the screen for the patients: if an „A“ appears with a green rocket the patient has to try to change their SCP in the positive (down) direction, if a „B“ appears the patients have to move it into the negative (up) direction. They know that negative SCPs increase seizure probability, positive SCPs reduce it. At the end of each training period, a larger proportion of positivity (70%) is trained in order to facilitate the transfer the suppression of seizures to he home environment (from Kotchoubey et al., Epilepsia (2001), with kind permission)

The drugs reduce the discharge of the affected neurons, preventing aura and seizure. Patients were trained to perceive their first seizure signs, expressed as electrically negative slow cortical potentials (SCP) of the electroencephalogram (EEG), and to lower the excitatory increase (voltage from negative to more positive). On a screen, they were able to observe these brain potentials in the form of flying “rockets” and use them to lower their neuronal excitation (see Fig. 2).

It required extremely long training periods of more than 50 h in these very severely ill people, which of course prevented the widespread use of this life-saving method in clinical practice. But the fact that almost all patients - even those with severe cognitive impairments - learned this brain strategy, 35% prevented their seizures completely, and almost all learned to reduce them, shows that learning to regulate the electrical brain processes to perfection like an athletic performance or a cognitive skill, is possible (Koutchoubey et al., 1996, 1999, 2001; Birbaumer and Kimmel, 1979; Rockstroh et al., 1989; Carlson, Seifert & Birbaumer, 1994). A ten year follow-up demonstrated significant seizure reduction and continuous capacity to self regulate SCP (Strehl et al., 2014; for a meta analysis of neurofeedback in epilepsy, see Tan et al., 2009).

The almost perfect performance in the psychological control of the brain electrical processes of these patients finally led to the application of BCIs in completely paralyzed people. While non-invasive neurofeedback training usually results in only partial control of the brain waves, here the extremely long training periods and session numbers may have resulted in such high responder rates. In addition, patients with debilitating disease such as drug-resistant epilepsy and complete paralysis may be highly motivated to carry the burden of excessive long training times, The reasoning was that with perfect or almost perfect brain control one could also select letters and sentences from a computer menu and operate switches with the learned brain potentials. Exactly that was then realized.

#### Rehabilitation after Stroke

Worldwide 1.6% of the population suffers a stroke within one year. From the age of 75, this figure rises to 6.5%. One third of those affected die within the first year after the stroke. One third to one quarter is so severely disabled that even after years of therapy and rehabilitation, purposeful movements of the affected paralyzed hand are not possible. It is for this treatment-resistant group that BCI is intended. The less severe patients recover differently usually after physiotherapy and or prolonged training at rehabilitation facilities with intensive exercise programs.

The vast majority of strokes occurs due to occlusion of a cerebral vessel (ischemia), a minority due to injury with bleeding of the cerebral vessel (hemorraghia) into the surrounding brain. Both lead to the death of the affected brain cells if the vessel is not opened within 1–4 h afterwards or if the injured leak is closed. Often vessels controlling the locomotor system of the opposite side of the body are affected, therefore unilateral paresis almost always results.

**Fig. 3:**
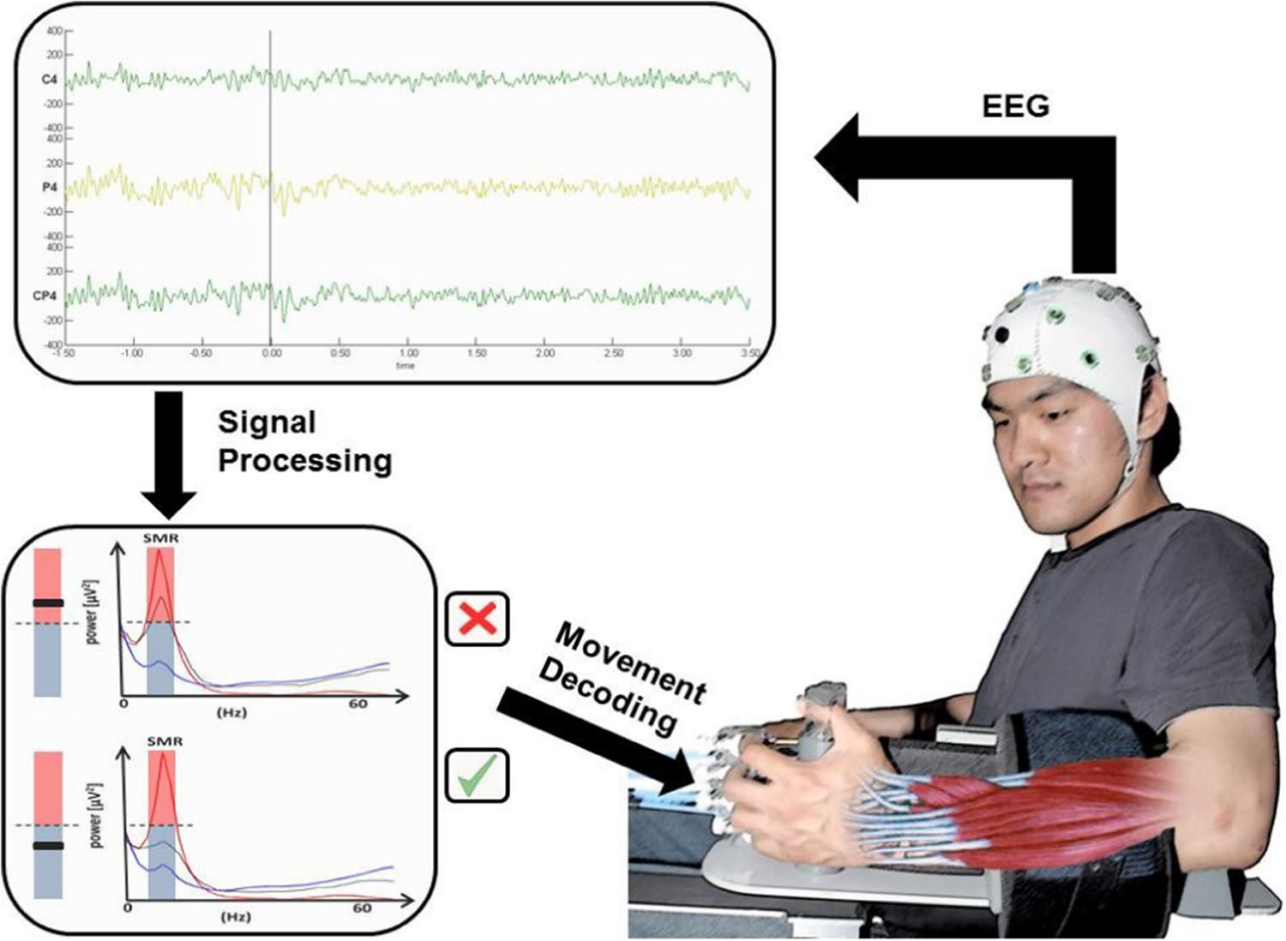
Experimental set-up of BCI in complete paralysis of the hand after unilateral stroke (from Ramos et al., Annals of Neurology, 2013, with kind permission). See text for explanation

Figure 3 shows the BCI as it was successfully used for the rehabilitation of total paresis (paralysis) of the hand and arm in a controlled study by Ramos-Murguialday et al. (2013). In this procedure, the paralyzed arm and/or hand of the patient(s) is fixed in an orthosis/prosthesis and they must try to move the arm, or at least imagine a movement, in response to a signal. If this succeeds, the simultaneously registered brain activity desynchronizes (the central mu-rhythm of 8–15 Hz is replaced by higher frequencies in the beta range) and activates the orthosis, which now moves the hand/arm on the imagined/imagined willful command (under the link in the publication of Ramos the reader can follow this in a video). This is repeated over many hours and hundreds of times. After each session, the same movement is practiced without BCI and without technical or BCI-support in a real life situation together with a physiotherapist.

The decisive advantage, which makes this method significantly more efficient than all previous rehabilitation attempts in completely unilaterally paralyzed stroke victims (in the case of existing residual movement, constraint-induced movement therapy (CIMT, see below) and physiotherapy is more appropriate), is the fact that the patient, or rather the patient’s brain, receives immediate feedback corresponding to the success of their act of volition: the brain associates a movement impulse with the successful consequence of this movement. This associative process connects the existing healthy neurons in the movement areas, which were previously silent and not activated, and leads to a reorganization of the brain in the vicinity of the destroyed areas, as the authors could see in the magnetic resonance images of the patients before and after BCI therapy and one year later (Ramos-Murguialday et al., 2019). The results of Ramos et al. (2013) were replicated in several studies; see Vidaurre et al., 2023). Especially the compensatory overuse of the healthy hand and corresponding brain hemisphere is replaced by the activation of the diseased brain hemisphere. Patients without any residual movement after years of classic rehabilitative treatment such as physiotherapy, robotic treatment and non-invasive brain stimulation responded in those BCI-studies with significant improvements of varying effect sizes, thus clearly demonstrating the clinical usefulness of BCI-Treatment in combination with physiotherapy (for a review see Vidaurre et al., 2023).

It will still take some years until the expensive rehabilitation and therapy measures for these severely affected people will be replaced by BCI. The human brain, especially that of specialists, needs a long time to replace entrenched behaviors and therapeutic measures in the health care system, unless immediate necessity or compulsion accelerates this.

### Communication in Locked-in-Syndrome (LIS)

In the literature, there are testimonies of people who, mostly after a deep subcortical stroke, were completely paralyzed and could only answer questions or signal their needs with eye movements or muscle twitches in their faces: for example, the grandfather in Dumas’ “Count of Monte Cristo” and the self-testimony of Jean Dominique Bauby, who dictated by means of eye movements after a stroke. His fate was shown in the film “Butterfly and Diving Bell” (1997), which drew worldwide attention to the tragic condition of those affected. The patients are awake and fully conscious. They understand speech, but cannot speak. A harrowing book and corresponding movie by Dalton Trumbo “Jonny got his gun” describes the fate of a soldier who lost all his limbs in World War I and could not speak, hear or see but had an intact thinking apparatus. Finally, Edgar Allan Poe, in several stories, brought buried and walled-in people to life or made them witnesses and triggers of terrible crimes.

However, no one until recently has yet obtained a coherent self-testimony of a person who was completely locked-in, i.e. could not give any signal - not even by eye movements - to the outside world, although mentally intact and fully conscious. This state is called “Complete Locked-in State (CLIS)”. CLIS occurs primarily in the advanced stages of amyotrophic lateral sclerosis (ALS), also known in the U.S. as Lou Gehrig’s disease, after a popular baseball player who contracted it. We used BCI technology to establish communication with CLIS patients based on decades of neuropsychological and neurophysiological research and development and interaction with CLIS patients and their families (Gallegos-Ayala et al., 2014).

CLIS patients suffering from ALS lie on their back in bed for months to years and decades until the end of their life, are artificially fed and artificially ventilated (even breathing movements and swallowing is no longer possible). Their eyes are usually closed, now and then a caregiver or family member opens their eyes. However, these cannot remain open for long, otherwise they dry out without blinking or eye movements. Therefore, the ability to see is limited after prolonged illness. However, the sense of hearing, touch and pain are normal but they can only be tested with a BCI, since the person in CLIS cannot react. Thinking and feeling are also most likely normal, at least in the early period of the CLIS condition. Thinking and feeling too can only be tested with a BCI. The paralysed person experiences the world, hears the conversations of his/her environment, cannot react and therefore seems unreachably brainless.

As long as the eye muscles function or any other muscle of the body can be activated, communication with commercial computers is possible: by eye movements, pupil movements or blinking of the eyelid, which are registered of the computer via cameras or muscle potentials (“eye-tracker”), it is possible today to have letters and words selected quickly and, after some practice, without errors; sentence completion programs based on AI- algorithms recognize in a flash what the patient wants to formulate and facilitate communication.

Surprisingly, in all ALS patients who are in LIS (locked in state with eye-movements intact) or CLIS and are cared for by the family, the perceived quality of life is good. However, in CLIS, only a few patients have been surveyed with a BCI installed in the patient’s home for an extended period of time.

Only two examples of CLIS patients will be described here to illustrate the procedure with BCI (the patient W.F. reported here and the patient F. S. can be viewed in the videos of our publications (e.g. Gallegos-Ayala et al., 2014; Chaudhary et al., 2016, 2022).

#### W.F

Mrs. W.F., born in 1945, lived in Hamburg, Germany and was married. She had three children and several grandchildren. There had always been and continued to be close family cohesion, although one of the children lived in Spain after years of family residence in the Dominican Republic. The husband of Mrs.F. was a successful businessman and had operated a private airline in the Dominican Republic, his wife a fashion boutique in Santo Domingo. Around 2000, the family returned to Hamburg. As is so often the case with amyotrophic lateral sclerosis, calf cramps first appeared, then walking problems with signs of paralysis, which in the course of a few months forced Mrs. F. into the wheelchair. The husband ended his professional career to care for his wife. The diagnosis of ALS was made, after several misdiagnoses in 2006 by two medical centers for motor neuron disease (MND). The catastrophic course was described by the doctors and the patient and husband were advised to refuse invasive artificial respiration when respiratory problems begin, occuring in ALS from a certain stage of the disease, which of course means death (usually by suffocation). This is also typical for the way the majority of the medical profession deals with ALS patients. Psychological help was not given. The quality of life with permanent artificial respiration and artificial nutrition (neither breathing nor swallowing is possible in the advanced stage of ALS) was and still is presented as poor to catastrophic (this is incompatible with the scientific facts, but corresponds to the prejudice of most people involved with ALS). In a family council in 2007, involving the husband and the 3 children, it was decided to preserve the life of the mother in any case, against the advice of doctors and public opinion and against social pressure. J., the husband, together with a nurse from Cuba took care of his wife day and night. He also stayed awake at night when no nurse was present to monitor breathing, transfered his wife in a wheelchair and with a respirator every day to the surroundings in Hamburg, traveled with her in his van to their daughter in Spain, etc. until her death in 2021. The children supported whenever they could. In 2009 she could only move her eyes to select letters, in 2010 the family lost contact with her, the eye muscles and all other striated muscles of the body failed to work, she was in CLIS. Two year after that in 2012 the husband learned from an article in the press and on the internet about our attempts to restore communication in locked-in and completely locked-in patients (Birbaumer et al., 1999). However, only locked-in patients were described there. From August 2012, we were trying to communicate with Mrs. F. and her husband until her death with varying success.

The first year we tried with the help of a BCI we had described in Nature, which used slow electrical brain potentials in the electroencephalogram as communication signals. The experiments are always similar, no matter which brain signal is used for the BCI:

After the patient receives the electrodes (for EEG) or the infra- light sensitive sensors (for near infrared spectroscopy, NIRS) attached to the head and above and below the eyes (for eye movements, electrooculogram, EOG), they hear many general and personal questions, all of which require a yes or no answer and the answer is known to the patient, the family of the patient and the experimenters: e.g.” Berlin is the capital of France”, “Berlin is the capital of Germany”. The patient receives the instruction “to answer in the mind with yes or no”.

During this time, the computer, using an algorithm from artificial intelligence theory (most often so-called “Support Vector Machines”, SVM are used), tries to separate the imagined „yes“ from the imagined „no“ from the brain signal after the question has ended for about 25 s. After 40 to 60 such questions, the computer has usually (if the patient is not asleep or unconscious, which can be recognized by an experienced EEG specialist from the slow EEG) classified the answers in 70% or more correctly from the course of the physiological signal, if the patient has thought „yes“ or “ no“. After that the patient receives again 20–40 such questions and after each question the computer answers to the patient whether it has recognized the answer of the brain as yes or no. If the computer cannot classify the physiological response it remains silent. Through the continuous feedback the person learns to adjust to the computer’s classification method and learns to produce the “correct” brain signal, making it easier for the computer to classify the answer as yes or no. Only when consistently over 65-70% of the questions with known answers have been answered correctly, open questions with unknown answers are asked such as “Does your back hurt?” or “Do you want to be turned?” “Are you depressed?” with the respective counter questions („you are happy“, „your back does not hurt“etc.) for control.

These questions are always repeated on different days. With Mrs. F. and her husband we worked until her death, in the last years the husband was connected with our engineers electronically, operated the BCI alone, when he wanted to know something important from his wife. Experimenters and researchers have to become a friendly part of the family, as always with interactions over such long periods of time and intimate cooperation at the homes of the patients. Without such a close positive relationship a success of BCI-communication is unthinkable: this was often considered scientifically and politically unacceptable and in times of “political correctness"or „scientific neutrality“ sharply attacked. Questions about quality of life and inner distress and also questions about the desire to continue living require a close trusting relationship between researchers and the families of those affected. A “neutral-objective” attitude is counterproductive and prevents any success of this technology. Mrs. F. could not achieve a sustained communication result above 50% chance probability for the first few months with EEG as a BCI measure, therefore we measured cerebral blood flow in the anterior brain areas using infralight sensors (optodes) on the head with near infrared spectroscopy (NIRS). In NIRS, a infrared light penetrates through the skull onto the top layer of the brain and, with more blood flow, reflects fewer light quanta absorbed by hemoglobin, which is measured by sensors distributed next to the light sources on the head. Finally, the responses were reliable even over many years and could be published in a scientific journal (Gallegos-Ayala et al., 2014).

#### F.S.

F.S. was born in 1985, into a well-off German family, his sister J., older than him, is now the person legally responsible for the sick F., along with his wife L. Both have devoted themselves for years to care for the now completely paralyzed F. in his home environment. After attending school and graduating from high school, F. successfully studied economic management and worked in a good position.

In 2014, he complained of first limitations in his ability to move, twitching of the muscles and restrictions in sports and walking. It quickly got worse, speech difficulties were added, several neurologists were visited without success; until Professor Albert Ludolph in Ulm, Germany, pronounced the bitter diagnosis of ALS and informed F. and his family about the consequences and the course of the disease. However, F,and his wife did not give up, his wife even became pregnant and their son G. is the joy of all, especially of course for F. Together, they decided in 2016 for F. to have a tracheostomy when breathing became difficult and suffocation was imminent, and thus to continue living with the progressive paralysis. At this point most of those affected give up and refuse artificial ventilation and nutrition.

F. did not give up, until today, in a completely paralyzed state, he answers with the BCI to questions about quality of life, that he likes to live, loves life (his family), does not suffer from depression. This is consistent with most of our 50 or so patients who are cared for at home and are completely paralyzed (Lule et al. 2014, 2019; Clausen et al., 2017; Chaudhary et al., 2021). Since speaking also became impossible in 2016, F. received an"eye-tracker”, a computer that can be operated by movements of the eyes, which the computer detects via a camera. The computer offers the letters in the frequency of the alphabet and the patient can select or reject the letter with a short movement of the eye in one direction. After some time the computer automatically completes the word or sentence after the first letters (Tonin et al., 2020). F. could use the eye-tracker very well, but his eye movements became weaker and weaker and finally, in 2017, no one could recognize any movement and even opening and closing the eyes was possible only with external help. The family and of course F. were desperate and they contacted the author.

The encounters with F. and his young family were enjoyable and endearing from the beginning. The little son climbed around on his completely paralyzed and ventilated dad, watched TV with him, although F. almost only heard but saw little due to the eye paralysis, played at his feet. The nurses helped with breathing and hygiene and his wife Lena supervised everything with strict but loving attention, as is necessary with such patients 24 h a day: one wrong move, one forgetting to suck mucus, one single uncleanliness can mean suffocation or infection with life threatening symptoms and even death.

During our first visits we noticed that from time to time that Lena could still read eye movements of her husband, although neither the eye-tracker nor we could detect any movement. Therefore, we developed a BCI system that could also detect micro-movements of the eyeballs that could no longer be measured by the standard methods. For this purpose we used the changes of voltage of the electrically charged eye (positive at the front, electrically negative at the back of the retina). F. received his letter board again, but presented acoustically, because completely paralyzed ALS patients lose visual acuity when in late stages the eyes dry out due to lack of blinking and they cannot fixate due to eye muscle paralysis. F. should at least try to answer questions by intentional eye movements with “yes”. Although he could no longer select letters, he was able to answer important questions with yes for a few weeks (Tonin et al., 2020). But we all knew that this was only of limited duration, so we contacted a young neurosurgeon, Jens Lehmberg at a University hospital in the nearest town.

It was clear at this point that F. could not communicate even with the micro- eye movement system, neither with the BCI using electroencephalography (EEG) and near infrared spectroscopy (NIRS), all attempts were unsuccessful. The only solution left was the invasive one, the BCI had to be implanted into his brain, this had worked well for some ALS patients who were not yet completely paralyzed and could communicate with their eyes.

Donoghue (see Birbaumer and Hochberg, 2018) in particular had developed this technology, advised and counseled the team and co-authored the resulting paper (Chaudhary et al., 2022). Our neurosurgeon was enthusiastic, but advised us that only one neurosurgeon had sufficient experience with this procedure. We therefore bought the head of a deceased man in the USA, which was sent to us frozen, and asked that neurosurgeon who had successfully performed these operations in the U.S. and was now practicing in Cyprus, an Austrian from Graz (Gerhard Friehs) to “practice” with us and especially Jens Lehmberg. Gerhard Friehs was convinced of Jens‘ talent and competence after a successful simulation of the operation under operating room conditions. The young surgeon’s skill and care in implanting the 120 tiny microelectrodes into the central cortex was obvious, and we were able to begin the arduous process of obtaining governmental, medical, and legal approval after the responsible ethics committee of the hospital agreed: it was perfectly clear to the colleagues in the committee that there was no other option for Felix and that he should not be isolated from his outside world forever. The German Federal Institute for Drugs and Medical Devices (BfARM) gave its approval after intensive discussions and checks of the material, surgical and medical procedure. Of course, Lena and her sister Juli, who are legally responsible for Fe., had to give their consent despite all the risks (especially the risk of infection is particularly high during such procedures). F. himself had already given his consent before with the eye -BCI. Almost regularly, with such intensive and almost daily contacts over the years, a strong relationship of trust and usually also friendship develops with the families, as is the case also with F. and his family.

In March 2019, F. had already been unable to communicate with any system for weeks, and in a difficult operation lasting several hours, our surgeon, with his team and our help, implanted two micro-arrays Neuroport (Blackrock Neurotech), each containing 62 electrodes and measuring 8 × 8 mm in circumference, into two areas of his cerebrum: the so-called primary motor hand area and the area 2 cm in front of this area, the supplementary motor area (SMA). This was particularly important because the SMA also serves cognitive brain processes, intentions, expectations, etc. Afterwards we all shared with Jens the relieve and exhaustion, the intervention succeeded without any negative health consequences for F.

**Fig. 4:**
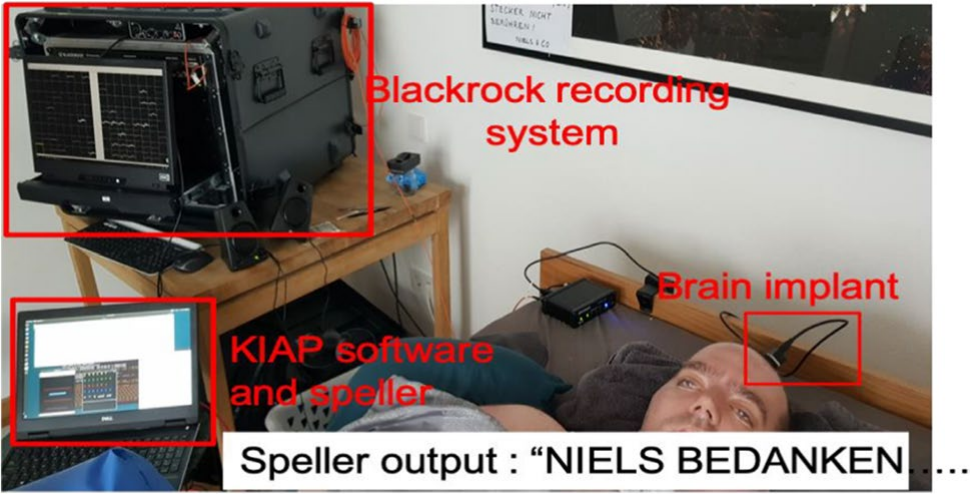
Experimental setup of the invasive Brain Computer Interface (BCI): Top left the computer, which converts the brain signals into numerical values and passes them on to the output computerscreen and loudspeaker and letter transformer (speller) (bottom left). On the right the patient F. with the implanted electrodes, whose signals are transformed by the implant to the processing computer unit on the top left

Already a few days after the operation, still in the intensive care unit, we tried to get in touch with F. via his implanted BCI. The “messages” from his brain cells from the 124 electrodes, from about 3 to 4 times as many neurons. Almost all electrodes worked and produced well identifiable signals, the neurosurgeon had done a good job. We asked Felix, when he wanted to say yes, to try to imagine something or to move his eyes in imagination as before. From the changes of the discharges of the neurons for yes and no to questions whose answers Felix knew, one should be able to analyze what means „yes“ and then later analyse and classify the same brain response at questions with unknown answer. We used computational operations and algorithms of “machine learning”. Over 84 days we continued our attempts with all kinds of experiments and images, without any tangible result and with increasing hopelessness. Should the „prophecy“, which was formulated after Aristotle’s theory of “thinking as movement " be true (see below and Birbaumer et al, 2012) The author always wanted to disprove an extinction of thinking and thought that was disproved myself long ago, because 4 completely paralyzed CLIS -patients had learned, after all, to at least say „yes and no” with the non-invasive NIRS-BCI (Chaudhary et al, 2016, 2021). Why should F. have suffered „extinction of thinking” as we had called it, after he had been closed off from the outside world for only a few months. He could not even reliably say “yes” with the implanted system! On the 87th day came the saving idea: We tried neurofeedback with Felix, as we had done for decades with epilepsy patients, scoliosis patients, stroke victims, etc.: We made the discharges of his brain cells from the SMA audible for him and asked him to influence the sound produced by these neural discharges himself. We told Felix to try thinking, because the imaginations had no effect (Try to tell, dear reader, how you can keep the balance of your bicycle! You cannot.) The vague term „thinking“ is often used in neurofeedback instruction with purpose to allow the patients to chose their idiosyncratic cognitive or behavioral covert strategy to control their brain activity. Already with the first attempt of self-control of the neuronal discharges he had success: as if “driven by a ghost hand” he controlled the pitch by increasing firing when he wanted to say yes. When he wanted to say no, he slowed down the firing. The key to success was neurofeedback! Since then, he has been able to select letters and communicate using his personal letter-spelling system. That day he “said” his first sentence with the discharges of his brain cells: “I thank Birbäumchen (that’s how he called the author of these lines) and his team”. Figure 4 shows the BCI arrangement at home as he has been using it for more than one year.

The result with F. is the final proof that a person in CLIS is able to communicate and participate mentally and emotionally in the life of their environment. This realization should fundamentally change the behavior of physicians, caregivers and visitors. It is the „duty“ of a society to care for these people and to keep them alive, just like any other ill person. Even if this is costly and time- consuming, we must ensure that these people can continue to communicate: the “extinction of thinking” does not take place. The success of the communication keeps thinking going.

### Theoretical and Ethical Consequences

#### Thinking and Movement

Probably Aristotle (384 − 322 BC) was not the first who described thinking and movement as “mutually conditioning”. However - as far as one can conclude from the translations - Aristotle avoided statements about the direction of the cause-effect chain: “…the contents of thinking are in the soul and they always have an approach or avoidance function, just like the actions. Soul is identical with movement, we can infer from a movement of the body a similar movement of the soul.” Similar statements exist in the fragments of the pre-Socratics, especially from Heraclit. The question remains unresolved and controversial to this day. Could it be that the question is asked with the wrong wording? Of course, the answer to this question depends on a correct (operational) definition of “thinking”. In most cases it is used as a synonymous expression for conscious language associated covert (internal) cognitive activity.

The “motor theory of thought” dominated experimental psychology at the end of the 19th and beginning of the 20th century and acquired substantial experimental evidence. This research reached ist peak with the book “Movement and Mental Imagery"of the first female doctor and professor of experimental psychology Margaret F. Washburn (1916). Washburn summarized a large number of psychological experiments that support her and Aristotle‘s theory that all thought originates from visible or invisible, sometimes extremly small, movement patterns.

If we follow Aristotle’s theory of the origin of thought (Aristotle equated the term “ anima“, i.e. soul with thought, although this is not so clear, or at least considered it related), he assumed that the direction of causality is from movement to thought and perception similar to what James (1890) and Lange (1887) postulated for feelings: the perception of peripheral muscular reactions, for example, flight determines the feeling of fear. Then, in the course of learning during human development, the direction of causality, at least subjectively, reverses; we experience our thinking as the cause, especially for voluntary movements. The assumed direction of causality would imply that in the absence or paralysis of movement in early development, thinking is learned from the effects of movements. This is precisely what the motor theory of thinking suggests and this is fundamental to our later analysis of the effects of motor paralysis on the thinking process. Extinction of thinking would be the inevitable consequence of paralysis from birth or before birth. The action-guided feedback from the musculature and the internal organs is understood in the motor theory of thinking and also in the James-Lange peripheral theory of emotions as the thinking-generating process.

However, before discussing the clinical and psychological consequences of *motor paralysis*, we must attempt to define or at least circumscribe the concept and process of thinking, distinguishing conscious (intentional) from nonconscious thinking, and pointing out that in the literature this is often referred to as controlled-intentional in contrast to automatic- unintentional thinking.The learning theories of the twentieth century, especially those of B. F. Skinner, have shown that volitional, linguistic, conscious thinking as we subjectively experience it, always forms a unity of action and its immediate positive or negative consequence, recognized in ancient philosophy as association and described there already as an elementary building block of the cognitive apparatus. We further assume in our definition that conscious thought (verbal, volitional) examined by Skinner as an “operant” always represents meaning. Neurophysiologically, meaning is generated through the synaptic connection of simultaneously discharging cortical neurons, as first described by the Canadian psychologist D. O. Hebb in 1949 and studied at the neuronal level by Singer (1993). With respect to our definition, it must be emphasized that non-conscious, automatic thought can also carry meaning. Let us take as an example the reflexive flight from a suddenly appearing snake many hundreds of milliseconds before the meaning of the snake is recognized as conscious. Already from this example it becomes clear that the predominant part of thinking processes takes place with and without meaning and not consciously. However, the sight of the snake nonconsciously already triggers the activation of the escape muscles, and the feedback from the periphery and muscles determines the thought.

**Fig. 5:**
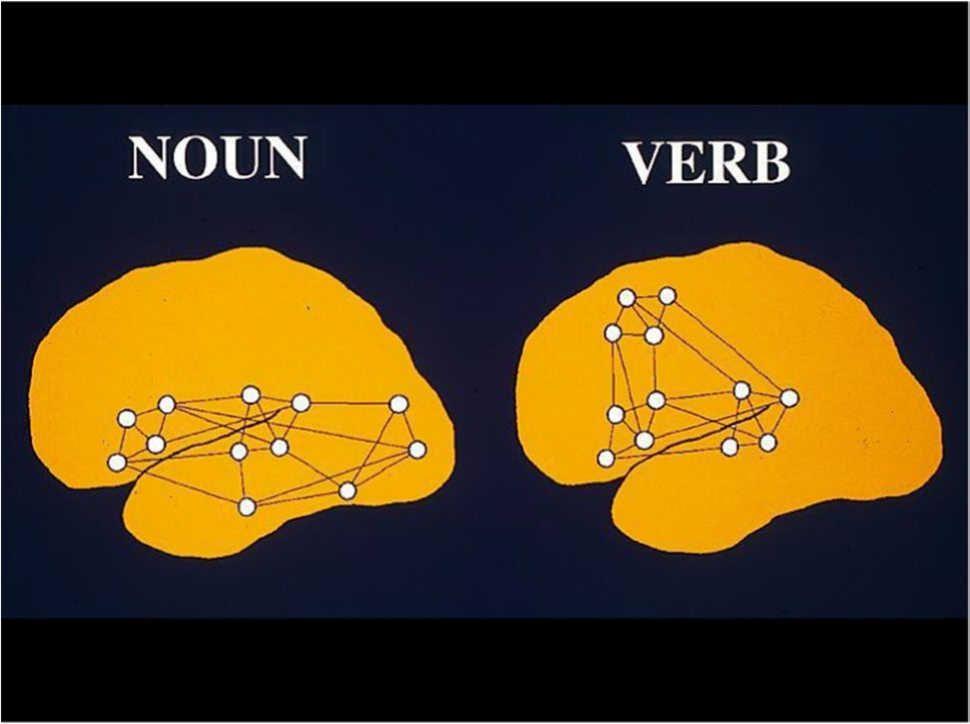
Schematic representation of two cell assemblies, on the left hemisphere for an object word, e.g. HOUSE, on the right for a verb, e.g. WALK: the associative and synaptic linkage takes place fronto–parietally for the object word and centro-frontally for the verb in cortical regions, where the corresponding visual (house) and motor (walk) ensembles are stored

An “operant”, i.e. the unity of action, consequence and meaning, can be illustrated by a hand movement also in dreamless sleep without external stimulus, caused by the discharge of nerve cells in the body, spinal cord or brain. The movement is “meaningless” and not directed toward a consequence; it is not remembered as a thought even when awake. Hebb called the neuronal basis of a thought with meaning a “cell-assembly”. Figure 5 illustrates this with the example of a cell-assembly for thinking a verb (for example, GO) and with the example of thinking a noun (for example, HOUSE) in the left cerebral hemisphere.

The Aristotelian question of our example from Fig. 5 is whether the thought (idea) HOUSE would have developed if it had never been associated with the movement of going in and out the house and the corresponding consequences. Let us use our example as a thought experiment: would the idea HOUSE ever arise in a person who spends time completely paralyzed in a house and had no concept of house (from before the paralysis) previously stored in working memory and would have left the house only passively lying on a stretcher, i.e., would never have performed the movements of entering or leaving the house? Would the sensory impressions, i.e., the perception of the house without any movement of one’s own, be sufficient to give rise to the concept and idea of house as a thought?

If we define a thought as an active and repeatedly occurring concept or perception based on movements, we would have to assume that a person completely paralyzed from birth would not develop a concept and thought of a house even after being passively carried in and out of the house.

Accordingly, no associative-synaptically connected cell ensemble should develop at the brain level of such a person. The pure perception of changes in the environment or body sensations is not sufficient to enable thinking and imagination of these perceptual impressions.

A possibly more intuitive thought experiment can be conducted on the perception and imagination of pain. Would a child who is injured for the first time and is completely paralyzed from gestation onwards be able to develop an imagination and a corresponding thought of the unpleasant pain stimulus if there had never been any escape or defense movement associated with it? Since this child, due to the paralysis, could also not transmit any kind of information about its pain to the environment, attention and other reactions of the social environment would not occur. This example is important insofar as until today it ha soften been assumed in medicine and also in the general population that fetuses and newborns do not feel pain and therefore do not need anesthesia and anesthesiological care. This conclusion was also suggested primarily by the absence of defensive movements, similar to what we often experience in patients in a locked in state or vegetative state.

However, these patients often report that they had intense pain after communication was restored with a brain-computer interface. These cases do not prove anything, however, since these patients had already had a long period in their lives before the onset of paralysis, and thus all the conditions for learning and remembering pain (pain memory) had been met.

The registration of the neurophysiological correlates of pain stimuli in newborns and the development of the fetal magnetoencephalogram (fMEG) showed that even before birth, intense physiological responses appeared in the corresponding brain areas in response to unpleasant stimuli, so that the presence of pain sensations was inferred. However, the question remains whether these ,,pain correlates” represent the idea and sensation of pain or only nociceptive reflexes without “pain meaning” and without accompanying emotional reactions. Contrary to the conviction of behaviorism and B.F. Skinner, we are dependent on the verbal communication of the persons concerned for the solution of the problem.

An intersting case of pain memory relates to phantom limb pain. The fact that phantom pain occurs after amputation of body parts even without their existence is not very enlightening, since it has been identified as a memory phenomenon in the brain and, surprisingly, phantom pain does not occur in the case of congenitally absent limbs and after using a functioning prosthesis that allows the entire volitional action to be performed normally and generating appropriate consequences of movement success (Flor et al., 1995). This means that the imagination and thought and memory of the pain has to contain the entire unity of pain stimulus, defensive movement, and consequences (i.e. social attention) for the imagination and thought to develop. Individuals who did not experience pain in the subsequently amputated limb before the amputation and during the trauma do not develop phantom pain (Flor & Turk, 2011). This would suggest that without execution of the movement sequence, for example, a paralyzed person does not develop the memory content (thought). The part of the body (arm or leg) that is not perceived during daily life, since it is stored in memory from past movement sequences, can be activated in imagination at any time. The study of phantom pains indicates to us that the Aristotelian theory might be correct.

### Learned Non-use: Extinction of Thought?

In the 1960s, the American psychologist Edward Taub (1966, 1973) conducted a series of studies on monkeys that are of great theoretical and practical-clinical importance to rehabilitation medicine and the motor theory of thinking. These studies led to one of the most consequential political conflicts with the animal welfare movement and is one of the many instances of misguided and harmful political conflicts with science and violation of the constitutionally guaranteed freedom of research. Because of alleged but ultimatiely unfounded violation of animal welfare, Taub had to stop his research and lost his position. Taub was able to show that when a purposeful movement was not performed in a deafferented monkey deprived of sensory feedback from the motor act, even if it could be performed, the animals no longer made hand movements, they behaved as if they were paralysed. Taub surgically severed all nerve fibers leading to the brain at the exit of the sensory nerves in the spinal cord, so that the animals could no longer perceive sensations from the arm and hand. The consequence was, as already mentioned, the complete “paralysis” of hand and arm, although all motor outputs were intact and the execution of movement would have been possible. The animals showed the same behavior as patients after a stroke with partial destruction of the capsula interna, in which motor outputs often remain possible but all sensory information leading to the cerebrum is blocked.

Patients are thus paralyzed on one side of the arm and leg, although they could certainly perform the movements. The affected side of the body is “ignored”, often patients make attempts to move shortly after the stroke, but they are unsuccessful because of the lack of body perception. The failure “punishes” the intention to move, the patient moves to the healthy arm and is rewarded for the successful movement of the healthy body part. Over time, this leads to exclusive use of that body part and learned-non use of the diseased body part. The patient is classified as “hemiplegic”.

Taub performed the following “therapeutic” procedure on the hemiplegic monkeys: He fixated the healthy arm with a sling for several weeks and “forced” the animal to bring the food to the mouth with the sick (“paralyzed”) arm, which succeeded after many failed attempts and thus rewarded the movement sequence and led to the re-use of the neglected body part. Philosophically speaking, the idea, the thought of movement was reactivated and the arm and hand regained their “meaning”. Applied to humans, this principle of “learned non-use” has been transformed into constraint-induced movement therapy (CIMT), which became one of the most successful rehabilitation principles in stroke treatment. Here, too, the healthy arm is fixed in a sling, thus motivating the patient to perform daily activities with the paralyzed body part. Taub also wanted to clarify our central question, to what extent these mechanisms become effective even before birth. He deafferented one arm of a fetus as described above and reimplanted the animal into the womb. This procedure led to the so-called scandal about the “silver spring monkeys” (60 years later Nikos Logothetis of the Max Planck Institute in Tübingen met the same fate for a much more harmless but equally important experiment). Thus, we do not know whether the congenital defect in Taub’s experiment would have led to recovery of the paralyzed arm after birth following constraint-induced movement therapy. This is relevant to interprete the asscociation between action and thought in the locked-in patients described here. Assuming that the idea of movement could be activated again after birth would argue for the reawakening of thought in the long-time completely paralyzed. As we will describe in the following sections, in our investigations of completely paralyzed ALS-patients, we may assume that without communication, in the course of several years, there is a “deletion” or partial extinction of thinking and imaginative processes. However, in the few completely paralyzed ALS patients, even after years of BCI-guided yes-no communication, we found that an improvement in the ability to communicate by selecting letters from a computer menu with brain waves and blood flow changes of the brain could not be achieved. Only one of our patients (see above patient F.) who had only been in a completely paralyzed state for a short time learned to communicate fluently with single neurons responses after surgical implantation of microelectrodes in his brain.

#### “The World as Will and Representation” (Schopenhauer, 1844): Self-control - Movement Control

Let us define self-control at the psychological level (behavioral level) as those processes which, in art-historical-religious terms, underlie the successful resistance in the “Temptation of St. Anthony.” In analogous behavioral performances of our everyday life, the abandonment or inhibition, the postponement of a response that leads to an immediate but small rewardin favor of a later bigger rewardin the future is achieved. The function of thinking in this situation is exactly this: to achieve postponement and interruption of direct input-output processes in favor of “purely” internal problem-solving interactions of stored, usually communicatively acquired (via imitation (mimesis) and imagination) stimulus-response-consequence connections (“I’ll wait until…”) of a more useful action in the future. At an anatomical level this requires prefrontal brain structures responsible for working memory performance (remember the aim) and memory access also requires parietal and temporal brain structures. Although the self-control capabilities of humans are naturally better developed and more variable compared to higher mammals according to cortex development, they become efficient depending on the intelligence of the individual. Stupidity is often defined by weak self-control abilities. In the course of the evolution of the brain development during the last millennia no increase of self-control capabilities and no increase of the corresponding cortical structures and neuroelectrical processes can be detected. Thus, the growth of technical problem solving and technology development is opposed by complete stability of the self-control possibilities. Our brain could not keep up with the control of its technical products, because it still allows only labile interruption of the immediate stimulus-response-sequence chains over short time periods and is still determined by immediately given stimulus-response-sequence connections. The biological reasons of these tight limits of our self-control capabilities are unclear, but in the early days of mammalian and human evolutionary development, long-term self-control performance was neither necessary nor useful for survival. Given their stability over millennia, with no evidence of evolution, genetic factors must be responsible for the stability of the limitations of self-regulation, modification of which is possible but rare. That epigenetic mechanisms would allow a better learning of self-control and could undermine the rigidity of evolution is a hopeful vision since the investigations of Paul Kammerer at the end of the 19th century. Kammerer had already shown in an article in Nature in 1900 that frogs who learned to mate outside the water could pass on the necessary protrusions of their limbs to the next generations. Colloquially and in research, we usually use the term and the factors responsible for self-control in a positive context. If an alcohol addict, faced with full bottles of wine, reaches for a glass of water instead of a bottle of wine and maintains this, we see this “achievement” as admirable and a sign of well-developed self-control resources. However, self-control occurs at least as frequently in socially negative contexts. When the German soldier, who is a caring, well-adjusted family man at home, kills an entire Jewish village, children, women, old people, etc., in the cruelest way possible, without a trace of of compassion (“are parasites”), this constitutes also a “self-control performance” of his previously learned pro-social behaviors: he has to suppress with considerable cognitive energy (prefrontal resource deployment in the brain) the prosocial behavioral tendencies learned so far, divert attention from these “moral” memory contents and focus on the new “command structures” in working memory (“Jews are parasites”). In a sociological context, Hannah Arendt described this inimitably in her book “The Origins of Totalitarism “. Self-control occurs just as much in negative social contexts in psychopathy, which we have discussed in detail elsewhere (Birbaumer, 2017). Persons with this character trait (approximately 3–5% of the population) do not need attentional effort to block (self-control) antisocial behavioral tendencies; they flow automatically-directly from the stimulating situation to the antisocial acts.

#### **“The Slippery Road”: Euthanasia and Democracy**

About 90–95% of the above described ALS patients and their families decide against life and life-sustaining measures, exceptions are, however, known in some countries such as Japan, Israel and also presumably Germany, where more patients decide for ventilation, perhaps because in these countries the care is good. However, especially in Israel and Germany, this may also be a response to the mass euthanasia under National Socialism. In Japan, on the other hand, there is a culture of respect for age and frailty, which perhaps explains the reluctance to euthanasia. The scientifically repeatedly shown high quality of life of ALS patients under ventilation has so far had little influence on the survival rate, especially in countries like Holland and Belgium with very “liberal” euthanasia laws. There, the number of those who are ventilated is much lower than in Germany, Japan and Israel, almost all choose death perhaps also related to social pressure.

With regard to this situation, it must be stated that euthanasia and assisted suicide should be justifiable and permitted by law under certain circumstances, although the limits should be much narrower than they usually are in existing laws. The only decisive factor must be the will of the persons concerned, who should express their wish to die several times at different times without the presence of other persons, only in the presence of a neutral recording device (without doctors, family members, etc.). It must be medically and above all in controlled scientific investigations proven that with prolongation of the suffering the quality of life remains extremely bad (which is often not the case with chronic illnesses such as ALS, Alzheimer’s disease, or multiple sclerosis or unipolar chronic depression, there the quality of life remains good after an initial decrease). No effective treatment should be available or about to be approved. The quality of life must be asked several times with the internationally calibrated questionnaires for quality of life and depression. A lawyer should determine that no financial interest exists, a psychologist has to provide a detailed behavioral analysis of all possible psychological and social influencing factors and a physician the improvement chance. Commercial suicide companies such as „Exit“ have to be excluded. In no case should a wish for euthanasia be fulfilled as in ALS, where it has been shown that the quality of life remains good and very good until the CLIS state, provided that loving care and nursing are available. If no communication with the patient is possible even with the use of BCI or other communication aids, for example in coma or vegetative state (non-responsive state), euthanasia must be avoided. Even after severe brain damage and persistent clouding of consciousness, improvements are possible and the quality of life cannot be determined in these states of altered consciousness.

Why is liberalization of euthanasia a threat to democratic forms of society? First of all, it is evident that the boundaries between murder and manslaughter and euthanasia (“killing on demand”) have historically been tighter in totalitarian forms of government and society than in democratic ones, although the liberalization of the last decades in Holland and Belgium and some states of the USA and Switzerland softened this historical fact. The mass euthanasia of sick or somehow different persons in Germany and Austria during National Socialism is the most gruesome example, although historical catastrophes such as these do not constitute empirical evidence for our thesis, and one can of course find examples of authoritarian rule that outlaw euthanasia and encourage murder and capital punishment, such as in the Mullah government of Iran and other countries at present.

In several publications we have presented data on quality of life (Lule et al., 2014, 2019; Chaudhary et al. 2016, 2021, 2022), which clearly show that even with many years of paralysis and even after acceptance of artificial nutrition and artificial ventilation, the quality of life remains good in most ALS patients and the desire for accelerated death decreases as the disease progresses. Nevertheless, most patients choose death before tracheostomy (more than 90%!). However, we and others have found that even in highly industrialized countries with well-developed medical systems, the knowledge of doctors, nurses and families of ALS patients about the good quality of life of the majority with such catastrophic disease progression is extremely incomplete and negative prejudices (“I do not want to live in this condition”) determine the decisions. In countries with liberal euthanasia legislation almost 100% decide against life, despite the data on quality of life! But what is valid for ALS, is possibly also valid for other diseases, which are accompanied by immobility and lack of communication in the late stages. People are often conscious but cannot express themselves motorically: we have found 30% of brain-damaged patients in “coma” or “vegetative state” (Kotchoubey et al., 2002) to be conscious while awake, the same is true for many late-stage organic and neurological diseases such as Parkinson’s, multiple sclerosis, tumors, etc. These patients are not able to express themselves motorically. These patients cannot express themselves without BCI, omission of assistance and active and passive euthanasia are nevertheless common, although no precise data exist. If euthanasia legislation is liberalized, the threshold for active intervention resulting in death without questioning the patients lacking communication will be lowered. With BCI or the above described “lie detectors” with registration of EEG or functional magnetic resonance imaging (fMRI, see Birbaumer et al. 2013) the threshold for euthanasia will increase. Only these psychophysiological measures and BCIs are able to ensure communication to critical questions for survival (Monti et al., 2010). In Germany, for example, we found that the best predictor of decision against life-sustaining measures was a positive response to the question, “Do you fear that your illness will be a burden to others?“. A certain role in the mass introduction of patients will these days have financial considerations about the cost of continuing life and care for the seriously ill and the critical situation of transplantation medicine with a shortage of transplants. In a democratic society, which supports the Helsinki protocols and the human rights charter of the UN, these financial considerations should not lead to a promotion of liberalization of euthanasia. This may end in liberalization related to „elimination“ of minorities and groups of people experienced as foreign (other ethnic background, other sexual orientation, persons with mental problems or intellectual diabilities or patients with rare debilitatiing diseases). From a legal, medical and moral point of view, the euthanasia of patients without communication, who may have a good quality of life, can be viewed as murder or at least manslaughter. All the more so, as the instruments to restore communication as described here exist and only the knowledge of their existence as well as the motivation to use them is missing.

## Conclusions

In order to advance scientific and clinical progress of invasive and non-inasive BCIs and neurofeedback for severe and otherwise untreatable clinical syndromes it is argued that learning principles in combination with a motor theory of thinking and cognition may serve as promising theoretical concepts for neurophysiological „mind-reading“ approaches, particularly in paralysis. In treatment-restistant epilepsy and chronic stroke without residual movement, non-invasive neurofeedback of cortical EEG-patterns and BCI-driven prosthetic devices have shown impressive results and need replication in larger controlled clinical studies. For locked-in syndrome and completely locked-in syndrome, particularly paralysis from ALS, non-invasive BCIs allowed simple „yes“ and „no“-communication with brain activity, while fluent selection of letters and words seem to be possible with invasive, brain implanted BCI systems only. The ethical and societal implications of communication with mind-reading devices are fundamental because they allow quality of life assessment in non-communicative persons in addition to their obvious quality of life improving clinical effects in seemingly hopeless medical conditions.

## Data Availability

No datasets were generated or analysed during the current study.
